# Confocal scanning laser ophthalmoscopy in glaucoma diagnosis and management

**Published:** 2010-08-25

**Authors:** C Alexandrescu, AM Dascalu, A Panca, A Sescioreanu, C Mitulescu, R Ciuluvica, L Voinea, C Celea

**Affiliations:** * >‘Carol Davila’ University of Medicine and Pharmacy, BucharestRomania; ** >The Opthalmology Clinic, University Emergency Hospital, BucharestRomania; *** >Central Military Hospital, BucharestRomania

**Keywords:** imaging technique, HRT, glaucoma

## Abstract

The early diagnosis and detection of progression are two key–elements in the actual management of glaucoma. The current opinion in clinical practice is to quantify the structural damage for a better follow–up of the patient and the standardization of the results. The present review is a concise survey of literature covering the period of 1990–2010, documenting the evidence–based role of confocal scanning laser ophthalmoscopy in glaucoma diagnosis and management

## Background

Glaucoma is one of the leading causes of blindness in the world. It is a chronic neurodegenerative disorder characterized by progressive loss of ganglionar retinal cells. Early diagnosis is essential for preventing the structural changes and irreversible vision loss. In this regard, literature of the last 20 years noticed the tendency to sustain clinical descriptive examination of the optic disc – subjective and prone to variability – by new objective imaging techniques, which provide a high reproducible quantitative assessment of the investigated structures and makes an inter–observer standardization of the results possible.

Confocal scanning laser ophthalmoscopy is a non–invasive investigation that has been used initially to receive three–dimensional images of the retinal surface in vivo. The commercial name of the device is Heidelberg Retina Tomograph (HRT). Two variants are used in current practice: HRT Ⅱ and HRT Ⅲ. It contains three modules: one for glaucoma – used for the analysis of the optic disc and peripapillary retinal nerve fibers layer, one for the retina – used for macular disorders and one for cornea. The present references will consider only the glaucoma module.

Heidelberg Retina Tomograph is designed to scan the retinal surface with a diode laser, which has a wavelength of 670 nm. The precision of the method is based on the principle of confocality. Only the laser light reflected from the focal plane, which focuses at the level of the diaphragm, is allowed to pass and it is registered by the detector. The laser light reflected from planes situated anterior or posterior to the focal plan, which do not focus at the level of diaphragm, will be eliminated.

The scanning process is vertical and horizontal, by multiple focal plans, generating a total of 64 sections in the coronary plan, of 384 x 384 pixels each. If the focal plane is moved to different depths along the optic nerve (z–axis) and further optical sections are acquired, the result will be a layered three–dimensional image (tomography). These sections are computer reassembled, making the calculation of the heights of different structures of the optic disc possible. A very short time for image acquisition is needed: only 0.025–0.032 seconds. The investigation may be performed through undilated pupils. 147000 independent measures of the height and variations of height at the optic disc surface are performed. The contour of the optic disc must be drawn. The integrated software performs a stereometric analysis within the area of the optic disc (drawn manually by the observer in case of HRT Ⅱ and automatically in case of HRT Ⅲ). The results are presented by stereometric parameters and topographic maps of the optic disc surface and the adjacent retina, which are very helpful in glaucoma diagnosis and management.

**Figure 1:**
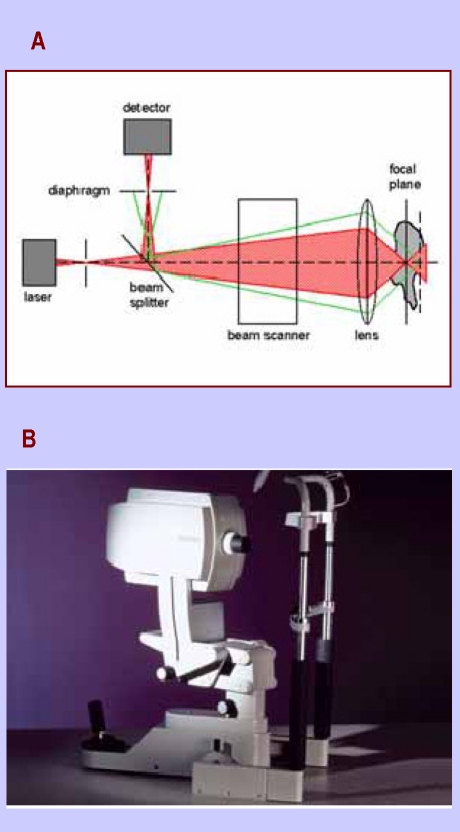
HRT  a) the principle of confocality; b) Heidelberg Retina Tomograph

## The display of the results

Once the optic disc contour is drawn, the software displays the stereometric parameters of the optic disc, which are compared to an interval of statistical ‘normal values’. All these parameters are calculated per total and per sectors of the optic disc, in relation to a reference plan arbitrarily generated by the computer software, which is situated at 50 micrometers (representing > 2 standard deviations of average segment height) below the mean retinal height measured at the disc border contour of a 6 degrees width infero–temporal segment (350–356 degrees). This sector, corresponding to the papillomacular bundle, is considered to remain the most stable in the course of glaucoma.

**Figure 2:**
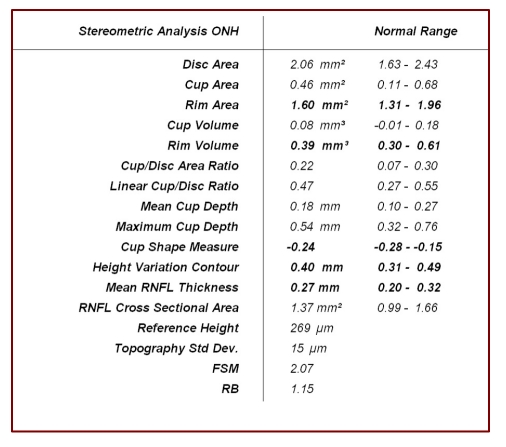
HRT Stereometric parameters of the optic disc

All the structures situated within the contour and above the reference plan are considered as neuroretinal rim by the computer analysis and the ones situated below the reference plan form the optic cup. One can notice that the correct drawing of the optic disc contour is very important in the calculation of the stereometric parameters. The corneal curvature also interferes with the measures and keratometry must be taken into account.

Another option for the display of the results is a **topographic map**, where every scanned point has a code of colors according to its height in relation to the reference plan – darker if it is elevated and lighter if depressed.

**Figure 3:**
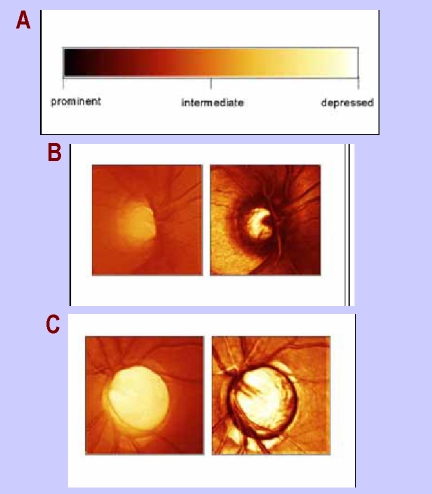
a) the code of colors; b) normal optic disc;  c) increased cupping in advanced glaucoma

Another important measure is made by the computer: the height variation of the peripapillary retina along the optic disc contour. The measure starts at the temporal level, then continues at the superior level, nasal, inferior and then temporal again. The normal aspect is of ‘double humps’, with 2 peeks, superior and inferior, where the retinal nerve fibers layer thickness is increased. In glaucoma, there is asymmetry or/and depression of the height variation contour, which does not reach the mean retinal height (MRH). 

Furthermore, two statistical analyses of the results are displayed by the HRT Ⅱ software: discriminant analysis and Moorfields Regression Analysis. 

**Discriminant analysis** uses a discriminant function (F), based on three most important parameters of the optic disc: cup shape measure, rim volume and retinal surface height variation. An eye is classified as normal, if F is positive and glaucomatous is F is negative. Clinical studies performed by Mikelberg and col. revealed a sensitivity of 87% and a specificity of 84% in the detection of the glaucomatous damage.

**Moorfields Regression Analysis (MRA)** is a linear regression analysis of rim area/disc area, using a prediction interval of 99% (Wollenstein and col, Ophthalmology 1998; 105: 1557–1563). The optic disc is divided in 6 sectors and each is classified as it follows: within normal limits (WNL), borderline (BL) and outside normal limits (ONL), as a result of comparison to an ethnic and age–specific database. In a group of early glaucoma patients, the method showed a sensitivity of 84% and a specificity of 96%. It has also been proved that MRA detects glaucomatous changes before perimetric defects appear, in a group of ocular hypertension subjects, thus identifying the ones which convert to glaucoma.

**Figure 4:**
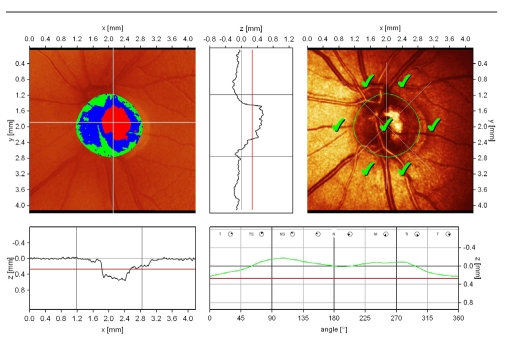
normal aspect of the optic disc
normal central cup, with shallow excavation, slight asymmetry because of the presence of vascular trunk;normal height variation contour value;low standard reference plane (red line);dynamic and symmetric height profile of the contour line, significantly above the mean retina height; The ‘double humps’ aspect is present;excellent reflectivity of the peripapillary retina;Moorfields Regression Analysis: within normal limits

**Figure 5:**
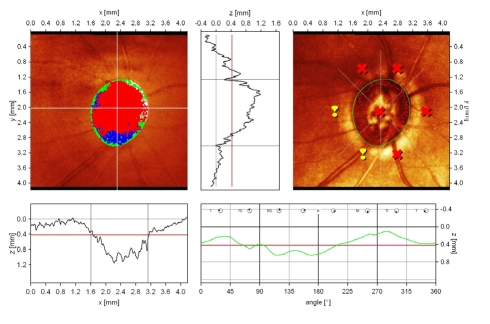
Advanced Glaucoma
large, deep cup, with significantly narrowed retinal rim;the excavation is increased both vertically and horizontally;flattened and asymmetric height profile of the contour line with depression in the superior sectorsboth polar peeks (‘double humps’) do not reach the mean retina height; reduced reflectivity of peripapillary retina

HRT Ⅲ software is improved with another model of statistical analysis and prediction: **Glaucoma Probability Score (GPS).**

Based on Swindale's technique, the analysis uses 2 measures of retinal nerve fiber layer (RNFL) on 2 meridians (vertical and horizontal) and 3 parameters of the optic disc (cup area, rim area, and mean cup depth) as input data and the software calculates the patient's probability to have glaucoma. The result is a score between 0% and 100%, interpreted as: 0–27%: WNL (within normal limits); 28–64%: BL (borderline); > 65%: ONL (outside normal limits)

**Figure 6:**
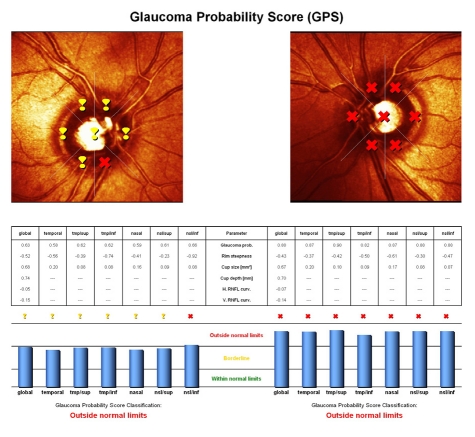
HRT Glaucoma Probability Score: RE: Borderline: global probability of glaucoma of 63%; LE: ONL: global probability of glaucoma of 88%

As the disc shapes and dimensions have significant variations among people, these methods of statistic analysis, based on cup/disc ratio, have a decreased sensitivity in case of an optic disc with anatomical particularities, where clinical judgment and the detection of changes in time are most important for diagnosis.

## Clinical relevance of HRT analysis

Reproducibility in terms of retinal surface height for each measured location is of 20 micrometers (BATHIJA and col., J Glaucoma 1998,121–127, Caprioli and col., Invest opththalmol Vis Sci 1998; 39:2321–2328). The coefficient of variation of the stereometric parameters is less than 5% (Mikelberg et col. J Glaucoma 1993; 2: 101–103, Rohrschneider si col Ophthalmology 1994; 101: 1044–1049). 

Short–term intraindividual variations in normal subjects are of less than 12%. Verdonck and col. revealed that among the stereometric parameters, the cup volume has the highest variability (up to 25%) and mean RNFL thickness has the lowest variability (1–3%).

## Detection of progression in glaucoma

Progression is present even in the actual definition of glaucoma. The identification of the minimal signs of change of the optic disc has a major impact upon the therapeutic algorithm and the quality of life of glaucoma patients. HRT is the only image technique that applies a rigorous statistical algorithm of analysis in order to differentiate the real biological change of test–retest variability. 

HRT Ⅱ and HRT Ⅲ Software display two methods for progression analysis:

- stereometric parameters analysis: quantitative global changes between 2 consecutive exams
- change probability maps: identify and localize the changes

Using the automated transfer of the optic disc contour from the baseline examination to the follow–up exams, the stereometric parameters may be easily compared in a time–related diagram. (Lesk and col, Ophthalmology 1999; 106: 1013–1018). 

As different parameters have different units of measure, a system of normalized changes has been developed, with limits between 0 no change and –1 if a normal eye converts to advanced glaucoma. Thus, the evolution in time of different parameters of the optic disc can be easily displayed on the same scale. HRT Software allows the comparison of data both globally and on different sectors of the optic disc. The most used are: temporal–inferior and temporal–superior octants, superior and inferior sectors and upper and lower hemispheres.

**HRT TCA (Topographic Change Analysis)** is an event–based technique that identifies the topographic variation in height of the optic disc elements. 384 x 384 pixels from the height variation contour of the optic disc are divided in 96 x 96 superpixels. An analysis of the height variation, which compares each follow–up examination with the baseline examination, is performed. The results are displayed in a color–coded map, from green – if there is elevation, to dark red – in severe depressed areas. In order to perform TCA 3 or more, consecutive examinations are necessary.

**Figure 7:**
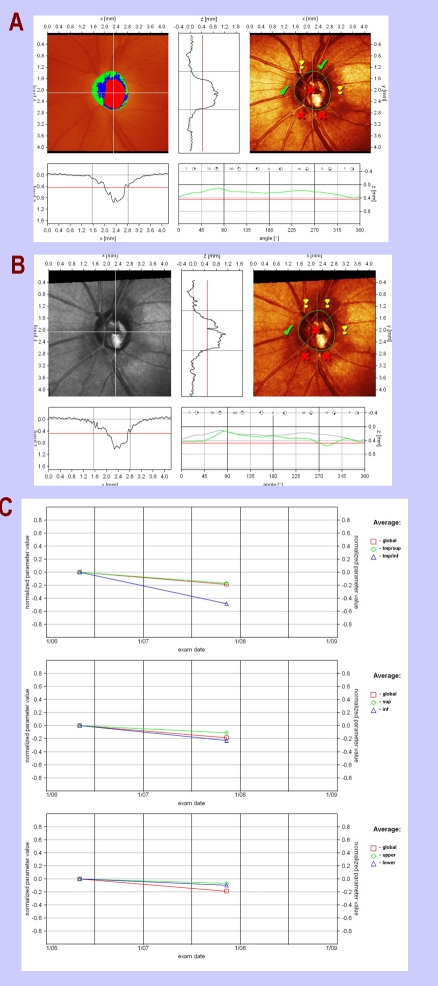
a) Baseline HRT; b) Follow–up HRT c) progression diagram:  a global progression of 0.2 (normalized value) in a follow–up period of 18 months, more severe in the temporal–inferior octant (0.5); height variation contour on both vertical and horizontal meridians shows the same result, with severe loss of neuronal fibers in temporal–inferior area

**Figure 8:**
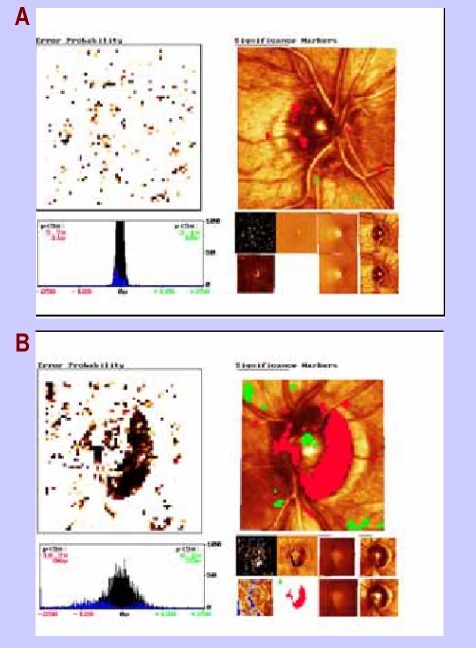
a) changes in heights are dispersed in some superpixels; there is no significant change; b) the progression of the glaucomatous disease is evidenced

A longitudinal study, made by Ichauchan and col., Arch Ophthalmol 2001; 119: 1492–1499), on a medium follow–up period of 5,5 years, evidenced that optic disc changes appear previous to those of visual field. In a long–term survey, this analysis showed the progression of glaucomatous damage in 70% of patients from the study group, and in 60% of those without losses of visual field. These observations proved that structural changes of the optic disc are more sensitive to progression. Detection of glaucoma progression is important both for the early diagnosis and for the management of glaucoma. Change may be the first sign of disease, especially in the case of optic discs with anatomic particularities, such as megalopapilla, micro disc or titled disc – often difficult to classify as normal or glaucomatous in the first exam. In evidence of a clinical significant change, early therapy can prevent further irreversible losses of visual field.

## Conclusions

 HRT Ⅱ and the new, improved version HRT Ⅲ offer high accurate, reproducible results, with good correlation to functional standard tests. Heidelberg Retina Tomograph can detect changes in the anatomy of the optic disc before visual field defects appear. Predictive value of HRT was confirmed by numerous clinical studies. Analysis of progression identifies the sectors with statistic significant changes and quantifies their evolution in time. These new instruments of computerized monitoring and analysis increase the confidence of the clinician in the detection of those changes of the optic disc that are biologically real and clinically relevant.

HRT Ⅲ Software is suitable for networking and telemedicine, as it permits the connection to the internet and the transmission of the results via email in .jpg or .bmp format. In this way, even the ophthalmologists who do not possess the device may visualize their patients' data of analysis.

Although further studies are needed to document its ability to predict and detect structural changes of the optic disc in time, HRT has been proved an important tool in the early diagnosis and management of glaucoma.
